# Six rehabilitation methods with acupuncture on consciousness in patients with traumatic brain injury: protocol for a network meta-analysis

**DOI:** 10.3389/fneur.2026.1737579

**Published:** 2026-06-19

**Authors:** Ning Sun, Yi He, Jing Xiong, Fang Xie, Chengqi He

**Affiliations:** 1 Rehabilitation Medicine Center and Institute of Rehabilitation Medicine, West China Hospital, Sichuan University, Chengdu, China; 2 Key Laboratory of Rehabilitation Medicine in Sichuan Province, West China Hospital, Sichuan University, Chengdu, China; 3 Acupuncture and Tuina School, Chengdu University of Traditional Chinese Medicine, Chengdu, China; 4 Department of Rehabilitation Medicine, West China Tianfu Hospital, Sichuan University, Chengdu, China

**Keywords:** acupuncture, consciousness recovery, disorders of consciousness, network meta-analysis, rehabilitation, traumatic brain injury

## Abstract

**Background:**

Disorders of consciousness (DoC) are common after traumatic brain injury (TBI) and are associated with prolonged hospitalization, high care needs, and long-term functional burden. In clinical practice, acupuncture is frequently delivered alongside other rehabilitation modalities such as repetitive transcranial magnetic stimulation, hyperbaric oxygen therapy, peripheral or cranial nerve stimulation, structured sensory stimulation, and Chinese herbal medicine. However, existing trials are small, heterogeneous, and rarely compared head to head; current evidence syntheses are largely modality-specific and do not provide a unified cross-modality comparative framework.

**Methods and analysis:**

The protocol of our systematic review and network meta-analysis (NMA) is reported following the PRISMA-P guidelines. We will search the following databases from inception to February 2026: PubMed, the Cochrane Central Register of Controlled Trials, Embase, Web of Science, CNKI, Wanfang Data, VIP, and SinoMed, as well as major clinical trial registries and gray literature sources. We will include randomized controlled trials enrolling adults with TBI–related DoC. Eligible interventions will be six rehabilitation modalities delivered in combination with acupuncture, and comparators will include the same modality without acupuncture, sham acupuncture, or usual care. The primary outcomes will be recovery of consciousness, measured as change in validated behavioral scales; secondary outcomes will include functional disability, neurological severity, mortality, and adverse events. We will conduct a Bayesian NMA in Stata (v15.1) and R (v4.1.3) using random-effects models, assess both global and local consistency, and evaluate small-study effects and publication bias. Interventions will be ranked using surface under the cumulative ranking curve, and certainty of evidence will be rated using the GRADE framework adapted for NMA.

**Discussion:**

This study will provide a comprehensive NMA of RCTs testing the added value of acupuncture across six rehabilitation modalities for TBI-related DoC. The synthesis will provide decision-ready, certainty-graded comparative evidence to guide practice, guideline development, and priorities for dosing, timing, and target populations.

## Introduction

Traumatic brain injury (TBI), caused by an external mechanical force to the head, is linked to high mortality and long-term disability (1). Global estimates indicate millions of emergency visits and hospitalizations each year (1). A recent global synthesis reports approximately 69 million new TBI cases annually (2). Incidence rises with aging populations and greater exposure to road traffic and falls, and is projected to increase over the next decade (3). Global Burden of Disease analyses show rising incident cases, substantial disability-adjusted life-year burden, and marked regional variation (3). Many survivors develop disorders of consciousness (DoC) (4). DoC is associated with prolonged hospitalization, medical complications, intensive nursing needs, and substantial caregiver and economic burden (5, 6). Persistent cognitive, behavioral, and autonomic disturbances further limit participation and delay community reintegration (1). Early and structured rehabilitation can improve arousal, reduce preventable complications, and support functional recovery (6). Clinicians therefore need clear comparative evidence to guide adjunctive rehabilitation choices (4). Authoritative practice recommendations for prolonged DoC emphasize standardized diagnosis and structured rehabilitative care, with the Coma Recovery Scale–Revised (CRS-R) recommended as a key behavioral assessment instrument (4, 7).

Acupuncture is frequently used as an adjunct in neurorehabilitation (8). Proposed mechanisms include modulation of thalamo-cortical and ascending arousal networks, regulation of autonomic balance and inflammatory responses, enhancement of cerebral perfusion, and facilitation of synaptic plasticity and large-scale network connectivity (8–12). In patients with TBI–related DoC, these effects could reduce the threshold for eliciting purposeful behavior and support progression to higher states of consciousness (13). In the broader field of post-traumatic DoC rehabilitation, multicenter placebo-controlled randomized evidence (e.g., amantadine) demonstrates that appropriately targeted interventions can accelerate recovery, supporting the need for robust comparative effectiveness frameworks (14). Acupuncture is relatively accessible and can be delivered alongside other rehabilitation methods.

Studies reports that acupuncture is combined with several rehabilitation modalities in DoC, including repetitive transcranial magnetic stimulation (rTMS), hyperbaric oxygen therapy (HBO), median nerve stimulation (MNS), transcutaneous auricular vagus nerve stimulation (taVNS), multisensory stimulation (MSS), and Chinese herbal medicine (CHM). Cortical priming by rTMS may interact with acupuncture-related plasticity, supporting a rationale for the pairing (15). Improved tissue oxygenation via HBO may act alongside acupuncture's reported anti-inflammatory and perfusion effects (16). Somatosensory and autonomic arousal inputs delivered by MNS and taVNS have been investigated in clinical studies in DoC, including randomized designs. These data provide a rationale to evaluate whether acupuncture adds incremental benefit when combined with these modalities (17–20). Enriched environmental input from MSS has documented benefits in acquired brain injury and DoC and can be integrated conceptually with acupuncture-induced network modulation (21–23). CHM may contribute neuroprotective or pro-recovery effects after TBI, complementing acupuncture-based rehabilitation (24). Nevertheless, most trials are small, heterogeneous in timing and dose, use varied control conditions, and rarely provide head-to-head comparisons (25).

Existing reviews are largely limited to pairwise and modality-specific syntheses, which restricts their applicability to clinical decision-making in this field. In routine rehabilitation practice, clinicians rarely make binary choices between a single acupuncture-augmented intervention and one comparator; instead, they must often choose among several competing adjunctive strategies, including acupuncture combined with rTMS, HBOT, MNS, taVNS, MSS, or CHM. Although pairwise meta-analyses can summarize direct evidence within individual modalities, they are unable to provide an integrated comparative framework across multiple strategies or to formally incorporate indirect evidence when head-to-head trials are scarce. Therefore, a network meta-analysis anchored on comparisons between the same rehabilitation modality with and without acupuncture is better suited to estimate the incremental contribution of acupuncture and to support cross-modality comparisons under prespecified transitivity assumptions.

## Methods and analysis

### Study design and registration

This study has been registered in PROSPERO with the identifier CRD420251177423. We will report in accordance with the PRISMA-P statement to maximize transparency and completeness (26). Our methodology will follow established best practices: procedural steps will reference the Cochrane Handbook for Systematic Reviews of Interventions, and the certainty of evidence will be judged using the Grading of Recommendations Assessment, Development and Evaluation (GRADE) framework (27). For the NMA, we will align with PRISMA-NMA recommendations (28); the work is scheduled to commence on 10 January 2026 and to finish by 20 May 2026.

### Eligibility criteria

#### Types of participants

We will include adult patients (≥18 years, no restriction on sex) who have been diagnosed with a DoC following a TBI. DoC will include states such as coma, vegetative state/unresponsive wakefulness syndrome (VS/UWS), and minimally conscious state (MCS), as defined by the original studies.

#### Types of interventions

Eligible interventions are multimodal rehabilitation approaches combining acupuncture with one of six adjunctive therapies: rTMS, HBOT, MNS, taVNS, MSS, or CHM. “Acupuncture” includes manual body acupuncture, electroacupuncture, and auricular acupuncture, and must stimulate recognized acupoints. For each included study, we will extract acupuncture details including technique, acupoints used, session frequency, session duration, total treatment course, and whether the protocol was fixed or individualized (including pattern differentiation if reported). For electroacupuncture, we will additionally extract electrical stimulation parameters when available (e.g., frequency, intensity, waveform, and stimulation duration). Recognized acupoints will be defined as standard, named acupuncture points described in internationally used nomenclature or equivalent national standards cited by the original trial. To ensure adequate characterization of the acupuncture exposure, eligible trials must report, at minimum, the acupoints used and core dosing elements (session frequency, session duration, and total treatment course). Studies that do not provide sufficient information to define the acupuncture intervention will be described qualitatively but will not be included in quantitative synthesis.

For the co-interventions, we will extract key dosing/intensity parameters as reported, including: rTMS (target region, frequency, intensity, number of pulses/sessions), HBOT (pressure, session duration, number of sessions), MNS and taVNS (device type, stimulation site, frequency/intensity/duty cycle, session duration), MSS (program components and schedule), and CHM (formula name, composition or key herbs when reported, dose, frequency, and treatment duration). We will also record the timing/sequence of acupuncture relative to the co-intervention (e.g., delivered within the same session, on the same day but separated, or in phased/sequential periods), when reported.

#### Types of comparators

Comparator conditions will be prespecified as distinct nodes to avoid mixing control conditions with different non-specific and expectancy effects. Specifically, eligible comparators include: (i) the same co-intervention delivered alone without acupuncture (e.g., rTMS alone, HBOT alone, MNS alone, taVNS alone, MSS alone, or CHM alone), with otherwise comparable dose and scheduling when reported; (ii) sham acupuncture (e.g., non-penetrating needles or needling at non-acupoints) delivered alongside the same co-intervention; and (iii) usual care/standard medical management, with or without the same co-intervention as defined by the original trial. These comparator nodes will not be pooled by default. Where pooling is considered necessary to achieve network connectivity, we will justify the decision a priori for the specific outcome and evaluate its impact using sensitivity analyses.

#### Types of outcomes

The primary outcome is change in level of consciousness from baseline to the end of treatment or to the earliest reported follow-up, measured using one of the following validated scales: Glasgow Coma Scale (GCS) (29), reported as total GCS score change; or CRS-R (30), reported as total CRS-R score change. We will accept either measure, recognizing that the GCS is more frequently applied in acute DoC after TBI, whereas the CRS-R is considered the reference standard for assessment in prolonged DoC. Recent critical care and rehabilitation studies further support the feasibility and sensitivity of CRS-R for monitoring recovery trajectories across care phases (31). We will analyze GCS and CRS-R separately as scale-specific primary outcomes. Pooling across scales will be restricted to sensitivity analysis only.

Secondary outcomes will include: Rate of transition to a higher state of consciousness; Change in the Full Outline of UnResponsiveness (FOUR) score (32, 33); Change in Disability Rating Scale (DRS) score (34, 35); All-cause mortality during the study period; Adverse events or safety-related outcomes attributable to the intervention.

#### Types of studies

We will include only RCTs. There will be no restrictions on publication language or study location.

### Exclusion criteria

We will exclude studies with participants <18 years or non-traumatic DoC; interventions without acupuncture at recognized acupoints or with insufficient reporting; non-randomized designs or non-original reports (e.g., abstracts without data, reviews, protocols, animal/mechanistic studies); or those lacking extractable consciousness outcomes (GCS, CRS-R, FOUR, DRS, or documented state transition).

### Search strategy

We will systematically search eight databases from inception to February 2026: PubMed, the Cochrane Central Register of Controlled Trials (CENTRAL), Embase, Web of Science, China National Knowledge Infrastructure (CNKI), Wanfang Data, VIP (Chinese Scientific Journals Database), and SinoMed (CBM). We will also screen major clinical trial registries (World Health Organization International Clinical Trials Registry Platform, ClinicalTrials.gov, Chinese Clinical Trial Registry) and gray literature sources (e.g., GreyNet International, OpenGrey, Google Scholar) to identify ongoing, unpublished, or otherwise non-indexed studies and reduce publication bias.

The search strategy will be developed using the PICOS framework and will combine controlled vocabulary (such as MeSH and Emtree) with relevant free-text keywords. We will search across three main concept groups: first, adult patients with DoC after TBI; second, acupuncture delivered; and third, study design terms indicating RCTs, such as “randomised,” “randomized,” and “controlled clinical trial.” Full database-specific search strategies are provided in Supplementary File 1. Strategies for other databases will be adapted to their respective indexing systems.

### Study selection and data extraction process

**Figure 1:**
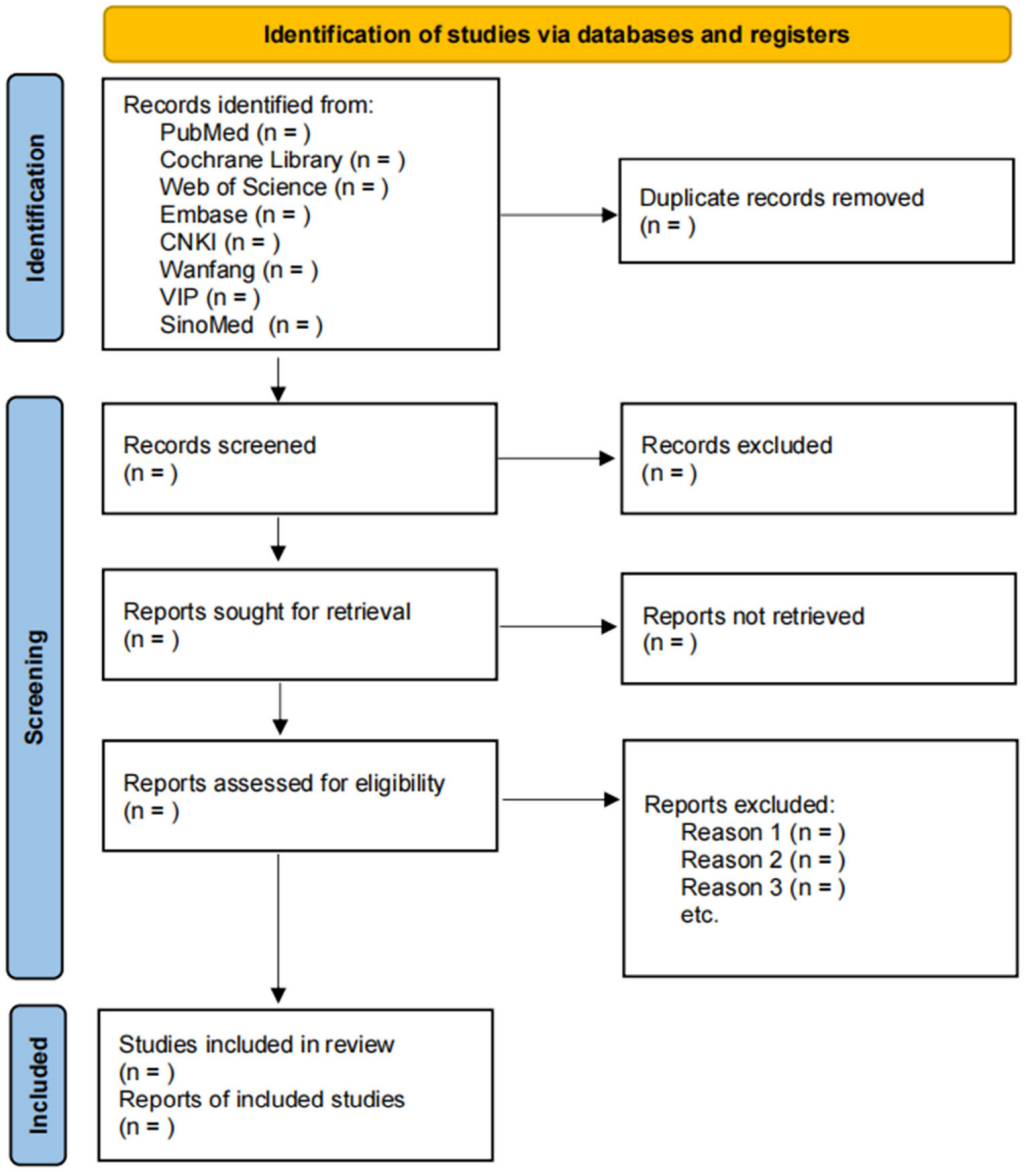
PRISMA 2020 flow diagram template for the planned study selection process. This figure is presented as a protocol template. The numbers at each stage and the reasons for full-text exclusions will be completed and reported in the final systematic review after database searching and screening are completed.

All retrieved records will be imported into EndNote X9, and duplicate entries will be removed. Two reviewers (NS and YH) will then independently screen titles and abstracts, followed by full-text assessment of potentially eligible studies, according to the predefined inclusion and exclusion criteria. Disagreements at any stage will be resolved through discussion. If consensus cannot be reached, a third reviewer will adjudicate. Reasons for exclusion at the full-text stage will be documented, and the overall study selection process will be presented in a PRISMA flow diagram (Figure 1). As this manuscript reports a protocol, Figure 1 is provided as a PRISMA 2020 flow diagram template. The final numbers at each stage and the reasons for full-text exclusions will be completed and reported in the final systematic review after searches and screening are finished.

Data extraction will also be conducted independently and in duplicate using a piloted, standardized extraction form (Supplementary File 2). We will collect: (i) study characteristics (first author, year of publication, country or region, and sample size per study arm); (ii) participant characteristics (age, sex distribution, time since injury duration, baseline level of consciousness, and major comorbidities); (iii) intervention and comparator details, including acupuncture technique (manual/electroacupuncture/auricular), acupoints used, session frequency/duration, total course, and whether acupoint selection followed a fixed protocol or individualized prescription (including pattern differentiation if reported); for electroacupuncture, stimulation parameters (e.g., frequency/intensity/waveform) will be extracted when available; for each co-intervention, key dosing/intensity parameters will be recorded (e.g., rTMS target/frequency/intensity/pulses; HBOT pressure/duration; MNS/taVNS device settings; MSS structure; CHM formula/dose); and the timing/sequence of acupuncture relative to the co-intervention (same session/same day/phased), when reported; (iv) outcomes and assessment time points; and (v) numerical data required for effect size calculation (group means, standard deviations or standard errors, change scores, event counts, and number analyzed). We will extract outcome data at end of treatment and at the earliest reported follow-up, recording the exact assessment time point as reported. If essential information is missing or unclear, we will contact authors and, when appropriate, derive estimates from available statistics with transparent assumptions; studies with non-recoverable key outcome data will be excluded from quantitative synthesis.

### The risk of bias assessment

Risk of bias will be evaluated independently by two reviewers (NS and YH) using the Cochrane Risk of Bias 2.0 tool (RoB 2) (36). After completing their individual assessments, the reviewers will cross-check each judgement. Any disagreement will first be discussed; if consensus cannot be reached, a third reviewer will arbitrate and provide the final decision. Each study will then be classified into one of three overall judgements: low risk of bias, high risk of bias, or some concerns. We will generate both tabular and graphical summaries of the RoB 2 assessments to present the risk of bias for all included studies in an interpretable and transparent way.

### Pairwise meta-analysis

Prior to conducting the NMA, we will perform conventional pairwise meta-analyses for all direct head-to-head comparisons identified in the included randomized controlled trials. These analyses will be carried out in Stata version 15.1. Between-study heterogeneity will be quantified in Stata using the I^2^ statistic and the estimated between-study variance (τ^2^). We will use a random-effects model as the primary analytical approach for all outcomes, on the basis that true effects are expected to vary across trials. A fixed-effect model will be applied only as a sensitivity analysis, rather than switching models according to an arbitrary I^2^ threshold (37). Effect measures will be selected according to data type and scale. For continuous outcomes measured using the same instrument across studies, we will calculate the mean difference (MD) with corresponding 95% confidence intervals (CI). When studies report the same construct using different measurement scales, we will instead use the standardized mean difference (SMD) and its 95% CI. For dichotomous (binary) outcomes, we will pool results as relative risk (RR) with 95% CI. For outcomes with sparse evidence, we will interpret heterogeneity metrics cautiously. Where feasible, we will report prediction intervals to reflect the expected range of effects in a future study. Small-study effects will not be formally assessed in pairwise comparisons with fewer than 10 trials because funnel-plot–based methods are difficult to interpret and prone to spurious asymmetry under sparse evidence.

### Network meta-analysis

We will conduct the NMA in Stata (v15.1) and R (v4.1.3) within a Bayesian framework. Pairwise meta-analyses will be performed using the meta and/or metafor packages, and Bayesian NMA will be implemented primarily using gemtc with rjags/JAGS as the computational backend. Network geometry and related visualizations will be generated using netmeta where appropriate. The exact package versions used will be reported in the completed review. Markov chain Monte Carlo methods will be used to estimate model parameters while accounting for within-study correlations in multi-arm trials to avoid double counting (38, 39).

For continuous outcomes, treatment effects will be synthesized as mean differences (MDs) when the same instrument is used across studies (e.g., GCS or CRS-R total scores), with 95% credible intervals (CrIs). Standardized mean differences (SMDs) across different instruments will be used only in sensitivity analyses to summarize overall change in consciousness and will be interpreted cautiously. For dichotomous outcomes, treatment effects will be expressed as relative risks (RRs) with 95% CrIs. All NMA models will use a random-effects structure to account for between-study heterogeneity across treatment comparisons (40).

**Figure 2:**
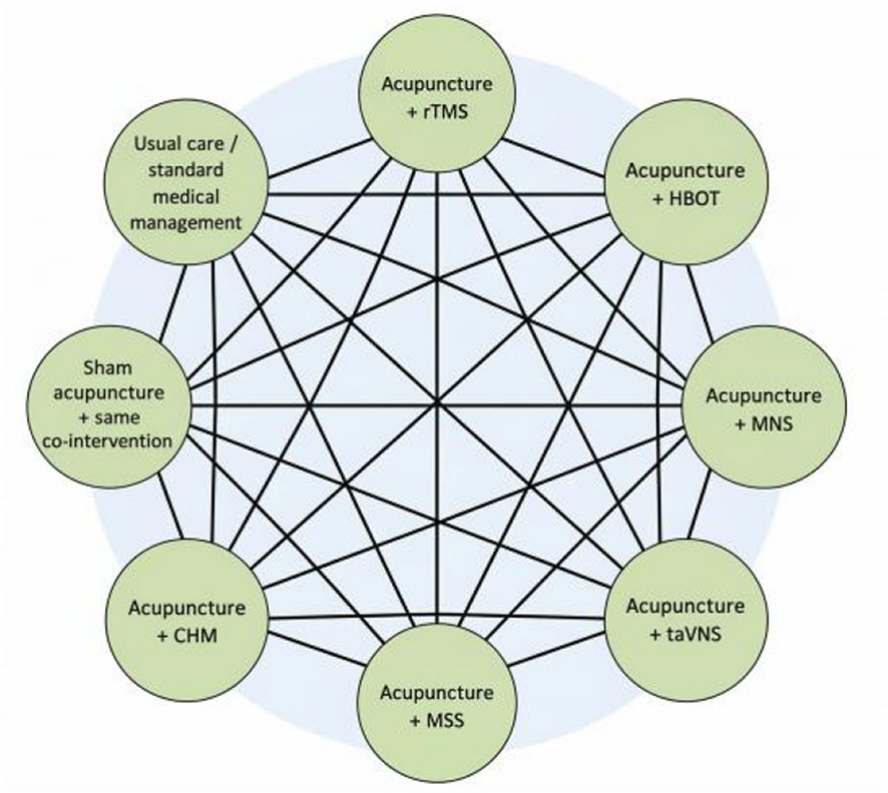
Schematic of potential comparisons between interventions within the network meta-analysis framework. rTMS, repetitive transcranial magnetic stimulation; HBOT, hyperbaric oxygen therapy; MNS, median nerve stimulation; taVNS, transauricular vagus nerve stimulation; MSS, multisensory stimulation; CHM, Chinese herbal medicine.

A schematic anticipated network structure is provided in Figure 2 to illustrate the prespecified intervention and comparator framework in this protocol. For visual simplicity, acupuncture technique is not further separated into manual acupuncture, electroacupuncture, and auricular acupuncture in this schematic figure; these will instead be examined as effect modifiers in subgroup analysis and network meta-regression where data permit. For each outcome, we will present the empirical network plot based on the included studies, with node size proportional to the total sample size and edge thickness proportional to the number of direct comparisons. We will also tabulate the number of studies and participants per node and per comparison, and report the number of connected components, so that readers can judge the extent to which each estimate is informed by direct and indirect evidence.

For the primary outcome-specific network, a treatment node will be retained only if it is informed by at least 2 studies or at least 20 participants in total and belongs to a connected component of the network. Nodes supported by a single very small study and not meaningfully connected to the rest of the network will not be included in the primary NMA; instead, they will be summarized narratively or, where feasible, by pairwise meta-analysis. If a network is sparse or disconnected, analyses will be restricted to connected subnetworks and/or direct pairwise comparisons. Isolated nodes will not contribute to indirect comparisons or treatment ranking in the primary NMA. In such cases, treatment rankings will be interpreted cautiously and presented only as supportive information alongside effect estimates and uncertainty.

We will use weakly informative priors for relative treatment effects and between-study heterogeneity (τ). Convergence will be assessed using trace plots and the potential scale reduction factor (R-hat). Model fit will be evaluated using standard Bayesian diagnostics, including residual deviance and, where appropriate, the deviance information criterion (DIC). To assess sensitivity to prior assumptions, we will re-estimate the primary models using alternative priors for τ and compare posterior effect estimates, CrI widths, and ranking probabilities. A material change will be considered present if alternative priors lead to any of the following: (i) a change in the direction of the posterior effect estimate; (ii) a change in whether the 95% CrI includes the null; (iii) a change of 10% or more in the posterior median effect estimate; or (iv) a change of 10 percentage points or more in surface under the cumulative ranking curve (SUCRA) or ranking probability. Any such changes will be reported explicitly and considered in the interpretation of robustness. All prior specifications and diagnostic results will be reported in the completed review.

Both global and local inconsistency will be assessed. Where data permit, local inconsistency will be examined using node-splitting or equivalent methods, and global inconsistency will be evaluated using model-based consistency diagnostics. Treatments will be ranked using the SUCRA and presented as rankograms, but rankings will be interpreted alongside effect estimates and certainty of evidence rather than in isolation. Where sufficient data are available, comparison-adjusted funnel plots will be used to explore small-study effects.

### Subgroup analysis and sensitivity analysis

Where data permit, we will conduct subgroup analyses to explore potential sources of heterogeneity. Prespecified subgroup variables will include time since injury (acute/subacute vs. prolonged DoC), baseline level of consciousness at enrolment (coma, VS/UWS, MCS), acupuncture technique (manual acupuncture, electroacupuncture, auricular acupuncture), co-intervention dose or intensity, age group, and overall risk of bias. We will also explore effect modification by the timing or sequence of acupuncture relative to the co-intervention and by acupoint selection strategy, where these data are sufficiently reported. For each subgroup, treatment effects on the primary outcomes will be examined using subgroup analysis, meta-regression, or network meta-regression, as appropriate.

For acupuncture technique, the primary approach will be to include technique as a categorical covariate in network meta-regression. Separate subgroup NMAs stratified by acupuncture technique will be conducted only if, within a given subgroup, there are at least 2 studies contributing to at least 1 connected comparison and at least 20 participants in total for the outcome of interest. If these criteria are not met, we will rely on the meta-regression results where feasible or otherwise summarize the findings narratively. These analyses are intended to reduce the risk of unstable or *post hoc* subgroup inferences under sparse data.

Sensitivity analyses will be performed to assess the robustness of the findings. Prespecified sensitivity analyses will include exclusion of studies at high risk of bias, comparison of random-effects and common-effect models, exclusion of trials with substantial missing or unclear data, and leave-one-out analyses to evaluate the influence of individual studies on pooled estimates and treatment rankings. If comparator nodes are combined to improve network connectivity, we will repeat the analyses using alternative comparator classifications to assess the robustness of effect estimates and rankings.

### Assessment of publication bias

Small-study effects and potential publication bias will be explored using comparison-adjusted funnel plots (Stata 15.1, netfunnel). This assessment will be conducted only for outcomes with at least 10 contributing trials because funnel-plot asymmetry is difficult to interpret and has low reliability when the number of studies is small. In addition, we will cross-check trial registry records against publications to identify potentially unpublished or selectively reported outcomes.

### Evidence quality assessment

We will evaluate the certainty of evidence using the GRADE approach (27), adapted for NMA. Two reviewers (NS and YH) will independently assess each outcome across five domains: risk of bias, heterogeneity/inconsistency, indirectness, imprecision, and publication bias. Risk of bias will be judged with RoB 2 at the outcome level. And we will downgrade if high-risk studies make a major contribution. Heterogeneity will be assessed using I^2^ and prediction intervals in pairwise meta-analyses, and the between-study variance in the NMA. Unexplained heterogeneity will lead to downgrading. Indirectness will be considered when there are concerns about population, intervention (including dose), comparator, outcome measure, or indirect comparison. Imprecision will be judged by the width of the 95% confidence or credible interval relative to a minimally important difference. Incoherence (disagreement between direct and indirect evidence) will be assessed with node-splitting and, if needed, global tests. Persistent incoherence will lead to downgrading. Publication bias and small-study effects will be examined using comparison-adjusted funnel plots and trial registry checks.

Each outcome will then be rated as high, moderate, low, or very low certainty. No downgrading corresponds to high certainty; one level to moderate; two levels to low; and three or more levels to very low. For outcomes informed mainly by indirect evidence or by sparse/poorly connected networks, we will consider downgrading for indirectness and/or imprecision as appropriate. When incoherence is detected, we will downgrade for incoherence and interpret treatment rankings cautiously. SUCRA rankings will be reported as supportive information and will be interpreted alongside effect estimates and the certainty of evidence rather than as stand-alone conclusions. Disagreements between reviewers will be resolved by discussion or by a third reviewer. All downgrading decisions will be recorded with brief justification. For the NMA, we will explicitly consider indirectness, incoherence, imprecision, and publication bias in GRADE ratings. If evidence is sparse, mainly indirect, or incoherent, we will downgrade certainty as appropriate. SUCRA rankings will be interpreted alongside effect estimates and certainty ratings, not in isolation. We will also consider indirectness when comparisons rely on differing comparator structures (e.g., sham acupuncture vs. usual care) and will downgrade certainty where such differences raise concerns about transitivity or applicability.

For indirect and network estimates, we will apply GRADE using prespecified downgrading rules. We will downgrade for indirectness or incoherence when effect modifiers differ materially across evidence or when node-splitting/global assessments show important unexplained disagreement, and we will judge imprecision and publication bias using credible-interval width and funnel-plot/registry signals with documented justifications.

### Protocol amendments

Any amendments or deviations from the registered PROSPERO protocol will be documented with the date, rationale, and affected section of the protocol. Major changes, including modifications to eligibility criteria, outcomes, comparator node definitions, statistical models, subgroup analyses, or sensitivity analyses, will be explicitly reported and justified in the completed review.

## Discussion

TBI-related DoC impose considerable clinical and societal burdens, yet routine practice often employs multimodal rehabilitation in which acupuncture is paired with rTMS, HBO, MNS, taVNS, MSS, or CHM without direct head-to-head comparisons across strategies. This protocol outlines a pre-registered NMA designed to compare each rehabilitation modality delivered with acupuncture against the same modality delivered without acupuncture, thereby estimating the incremental contribution of acupuncture and providing comparative effect estimates for adult TBI-related DoC. Treatment rankings, if generated, will be interpreted as exploratory and supportive rather than definitive, and not as stand-alone evidence for clinical hierarchy. By providing certainty-graded comparative estimates, this NMA is intended to complement existing international practice recommendations for prolonged DoC and to support decision-making where head-to-head evidence remains limited (4, 7).

A biologically plausible complementarity supports these combinations. Acupuncture has been linked to modulation of thalamo-cortical/ascending arousal systems, autonomic balance, inflammation, cerebral perfusion, and large-scale network connectivity (8–13, 23), which may interact with cortical priming from rTMS, oxygen delivery from HBO, afferent/autonomic drives from MNS/taVNS, enriched environmental input via MSS, and system-level support from CHM (15, 16, 19, 21–24, 41). Early clinical trials suggest potential synergy. Electroacupuncture plus rTMS improved mismatch negativity latency and GCS vs. rTMS alone (42); acupuncture plus HBO outperformed either therapy alone on GCS and response rates (43); acupuncture combined with MNS improved GCS, cerebral perfusion, and neurotransmitter indices without excess adverse events (44); and CHM plus acupuncture in TBI improved consciousness, function, cognition, cerebrovascular parameters, and gut-barrier markers vs. routine care (45). These data motivate a structured synthesis to quantify comparative effects and certainty.

To ensure interpretability and transparency, the review is registered and follows PRISMA with comprehensive bilingual searches, dual-reviewer screening/extraction, and RoB 2 risk-of-bias assessment. A Bayesian random-effects framework in Stata/R will model multi-arm correlations, assess global and local consistency, and explore small-study effects with comparison-adjusted funnel plots; SUCRA rankings will be treated as secondary to effect sizes and certainty (27, 38–40). By anchoring contrasts on “with vs. without acupuncture” for each modality, we aim to isolate acupuncture's added value rather than conflating benefits with greater treatment intensity.

We anticipate heterogeneity in injury chronicity (acute/subacute vs. prolonged DoC), baseline state (coma/VS-UWS/MCS), dosing parameters of both acupuncture and co-interventions, and outcome instruments. These will be addressed through prespecified subgroups and meta-regression, standardized mean differences where scales differ, predictive intervals, sensitivity analyses excluding high-risk studies, and leave-one-out influence checks. The synthesis is expected to identify the most promising acupuncture-augmented pairings and key effect modifiers, while delineating priorities for future multi-center, pre-registered RCTs.

Despite the strengths of comprehensive bilingual searches and prespecified analyses, several limitations warrant consideration. Clinical and methodological heterogeneity across trials is expected. This includes injury chronicity, baseline DoC state, intervention dose and duration, and outcome assessment timing. These differences may challenge transitivity and increase uncertainty even with random-effects models. The evidence network may also be sparse or poorly connected for some comparisons. This can increase reliance on indirect evidence and widen credible intervals. If incoherence between direct and indirect estimates is detected, we will interpret effects and rankings cautiously. Publication bias and selective outcome reporting may persist despite trial registry and gray literature searches. We will incorporate these concerns into RoB 2 assessments and reflect them in GRADE certainty ratings. Finally, correlated study characteristics and co-occurring design features may influence effect estimates. We will explore these factors using prespecified subgroup analyses, meta-regression where feasible, and sensitivity analyses.
