# Supplementary material for: Six rehabilitation methods with acupuncture on consciousness in patients with traumatic brain injury: protocol for a network meta-analysis
Source: Front Neurol. 2026 Jun 19;17:1737579. doi: 10.3389/fneur.2026.1737579 (PMC13333809; doi:10.3389/fneur.2026.1737579)
Supplement: Supplementary file 1 [file Table_1.docx]

**PubMed**

#1 Search: "Brain Injuries, Traumatic"[Mesh]

#2 Search: (((((((Traumatic Brain Injurie*[Title/Abstract]) OR (Brain Trauma*[Title/Abstract])) OR (Traumatic Encephalopath*[Title/Abstract])) OR (TBI[Title/Abstract])) ) OR (head injur*[Title/Abstract])) OR (brain injur*[Title/Abstract])) OR (neurotrauma*[Title/Abstract])

#3 #1 OR #2

#4 Acupuncture[MeSH Terms] OR Acupuncture Points[MeSH Terms] OR Acupuncture, Ear[MeSH Terms] OR Acupuncture Therapy[MeSH Terms] OR Auriculotherapy[MeSH Terms]

#5 Needling[Title/Abstract] OR Electroacupuncture*[Title/Abstract] OR Electro-acupuncture[Title/Abstract] OR Acupoint Therapy[Title/Abstract] OR Acupuncture Treatment[Title/Abstract] OR Acupuncture Treatments[Title/Abstract] OR Needle Therapy[Title/Abstract] OR silver needle[Title/Abstract] OR de qi[Title/Abstract] OR meridian[Title/Abstract] OR Auriculotherapy[Title/Abstract] OR needle pricking[Title/Abstract] OR needling[Title/Abstract] OR ear acupuncture[Title/Abstract]

#6 #4 OR #5

#7 ((Randomized Controlled Trial[Publication Type]) OR ("Randomized Controlled Trials as Topic"[Mesh])) OR ((((Randomized Controlled Trial) OR (RCT)) OR (Trial*)) OR (random*[Title/Abstract]))

#8 #3 AND #6 AND #7

**CENTRAL**

Search in Trials (Title/Abstract/Keywords)

#1 ("traumatic brain injur*" OR "brain injur*" OR "brain trauma*" OR neurotrauma* OR "head injur*" OR TBI OR "traumatic encephalopath*")

#2 (acupuncture OR electroacupuncture OR "electro-acupuncture" OR auriculotherapy OR "ear acupuncture" OR acupoint* OR needling OR "needle therapy" OR meridian OR "de qi")

#3 #1 AND #2

**Embase**

#1 ('traumatic brain injury'/exp OR 'brain injury'/exp OR neurotrauma*:ti,ab OR 'head injur*':ti,ab OR 'brain trauma*':ti,ab OR 'traumatic encephalopath*':ti,ab OR tbi:ti,ab OR 'traumatic brain injur*':ti,ab OR 'brain injur*':ti,ab)

#2 ('acupuncture'/exp OR 'electroacupuncture'/exp OR 'auriculotherapy'/exp OR acupuncture:ti,ab OR electroacupuncture*:ti,ab OR 'electro-acupuncture':ti,ab OR auriculotherapy:ti,ab OR 'ear acupuncture':ti,ab OR acupoint*:ti,ab OR needling:ti,ab OR 'needle therapy':ti,ab OR meridian:ti,ab OR 'de qi':ti,ab)

#3 ('randomized controlled trial'/exp OR 'randomization'/exp OR random*:ti,ab OR trial*:ti,ab OR rct:ti,ab)

#4 #1 AND #2 AND #3

**Web of Science Core Collection**

Topic = TS

#1 TS=(("traumatic brain injur*" OR "brain injur*" OR "brain trauma*" OR neurotrauma* OR "head injur*" OR TBI OR "traumatic encephalopath*"))

#2 TS=(acupuncture OR electroacupuncture* OR "electro-acupuncture" OR auriculotherapy OR "ear acupuncture" OR acupoint* OR needling OR "needle therapy" OR meridian OR "de qi")

#3 TS=(random* OR trial* OR RCT OR "controlled clinical trial")

¥4 #1 AND #2 AND #3

**SinoMed / CBM**

#1 主题/题名/摘要/关键词：(创伤性脑损伤 OR 颅脑损伤 OR 脑外伤 OR 颅脑外伤 OR 头部损伤 OR 神经创伤 OR TBI)

#2 主题/题名/摘要/关键词：(针刺 OR 针灸 OR 电针 OR 耳针 OR 穴位 OR 取穴 OR 得气)

#3 主题/题名/摘要/关键词：(随机 OR 随机对照 OR 随机对照试验 OR RCT OR 试验)

#4 #1AND #2 AND #3

**CNKI**

SU=(创伤性脑损伤 OR 颅脑损伤 OR 脑外伤 OR 颅脑外伤 OR 头部损伤 OR 神经创伤 OR TBI) AND SU=(针刺 OR 针灸 OR 电针 OR 耳针 OR 穴位 OR 取穴 OR 得气) AND SU=(随机 OR 随机对照 OR 随机对照试验 OR RCT OR 临床试验)

**Wanfang Data**

主题:(创伤性脑损伤 OR 颅脑损伤 OR 脑外伤 OR 颅脑外伤 OR 头部损伤 OR 神经创伤 OR TBI) AND 主题:(针刺 OR 针灸 OR 电针 OR 耳针 OR 穴位 OR 取穴 OR 得气) AND 主题:(随机 OR 随机对照 OR 随机对照试验 OR RCT OR 临床试验)

**VIP**

主题=(创伤性脑损伤 OR 颅脑损伤 OR 脑外伤 OR 颅脑外伤 OR 头部损伤 OR 神经创伤 OR TBI) AND主题=(针刺 OR 针灸 OR 电针 OR 耳针 OR 穴位 OR 取穴 OR 得气) AND主题=(随机 OR 随机对照 OR 随机对照试验 OR RCT OR 临床试验)
