# Supplementary material for: Six rehabilitation methods with acupuncture on consciousness in patients with traumatic brain injury: protocol for a network meta-analysis
Source: Front Neurol. 2026 Jun 19;17:1737579. doi: 10.3389/fneur.2026.1737579 (PMC13333809; doi:10.3389/fneur.2026.1737579)
Supplement: Supplementary file 2 [file Table_2.docx]

**Supplementary File S3. Standardized data extraction form**

Use this standardized form to extract study- and arm-level data for eligible randomized controlled trials (RCTs). Complete one row per study arm unless otherwise specified. Report data exactly as stated in the original article.

**A. Study identification**

| **Field** | **Entry** |
| --- | --- |
| Study ID (assigned) |  |
| First author |  |
| Year of publication |  |
| Country/region |  |
| Setting (ICU / rehabilitation / other) |  |
| Study design (parallel / cross-over / cluster) |  |
| Trial registration ID (if any) |  |
| Funding source(s) (as reported) |  |
| Number of arms |  |

**B. Participants (study-level; add arm-specific details if reported separately)**

| **Field** | **Entry** |
| --- | --- |
| Inclusion criteria (brief) |  |
| Diagnosis definition of DoC (coma / VS-UWS / MCS; as used by the study) |  |
| Time since injury at enrollment (days/weeks; acute/subacute/prolonged; as defined) |  |
| Age (mean±SD or median[IQR]) |  |
| Sex (n male / n female) |  |
| Key comorbidities or exclusions (brief) |  |
| Baseline consciousness scale(s) and score(s) (GCS/CRS-R/FOUR/DRS) |  |
| Concomitant care permitted/required (brief) |  |

**C. Intervention details (complete per arm)**

| **Field** | **Entry** |
| --- | --- |
| Arm label (e.g., EA+rTMS; rTMS alone; sham; usual care) |  |
| Acupuncture included? (Y/N) |  |
| Acupuncture type (manual / electroacupuncture / auricular) |  |
| Acupoints used (list) |  |
| Acupoint selection strategy (fixed protocol / individualized; pattern differentiation if reported) |  |
| Needle retention time (minutes; if reported) |  |
| Session frequency (sessions/week) |  |
| Session duration (minutes) |  |
| Total treatment course (weeks; total sessions) |  |
| Electroacupuncture parameters (frequency, intensity, waveform; if reported) |  |
| Co-intervention type (rTMS / HBOT / MNS / taVNS / MSS / CHM) |  |
| Co-intervention dose/settings (key parameters; as reported) |  |
| Timing/sequence with acupuncture (same session / same day / phased; if reported) |  |
| Comparator details (if sham/usual care; describe) |  |
| Treatment adherence/compliance (if reported) |  |

**D. Outcomes (complete per arm, per time point)**

| **Field** | **Entry** |
| --- | --- |
| Primary outcome measure (GCS change / CRS-R change; specify) |  |
| Outcome time point (end of treatment; earliest follow-up; exact day/week) |  |
| Outcome type (post-treatment value / change score) |  |
| Mean |  |
| SD (or SE/CI; specify) |  |
| N analyzed |  |
| Transition to higher consciousness state (events/total; definition) |  |
| FOUR score (mean, SD, N; time point) |  |
| DRS score (mean, SD, N; time point) |  |
| All-cause mortality (events/total; follow-up window) |  |
| Adverse events (events/total; description; attribution) |  |
| Other outcomes reported (list briefly) |  |

**E. Risk of bias (RoB 2; outcome-specific where applicable)**

| **Field** | **Entry** |
| --- | --- |
| Randomization process |  |
| Deviations from intended interventions |  |
| Missing outcome data |  |
| Measurement of the outcome |  |
| Selection of the reported result |  |
| Overall RoB 2 judgement (low / some concerns / high) |  |
| Notes/justification (brief) |  |

**F. Missing data and notes**

| **Field** | **Entry** |
| --- | --- |
| Missing/unclear data items (list) |  |
| Author contact attempted? (Y/N; date) |  |
| Derivation performed from available statistics? (Y/N; method) |  |
| Assumptions made (if any) |  |
| Additional comments |  |
